# Review of Development Survey of Phase Change Material Models in Building Applications

**DOI:** 10.1155/2014/391690

**Published:** 2014-09-11

**Authors:** Hussein J. Akeiber, Mazlan A. Wahid, Hasanen M. Hussen, Abdulrahman Th. Mohammad

**Affiliations:** ^1^Department of Mechanical Engineering, University Technology Malaysia, Johor Bahru, Malaysia; ^2^Machine and Mechanical Department, University of Technology, Baghdad, Iraq; ^3^Department of Mechanical Engineering, Baqubah Technical Institute, Foundation of Technical Education, Baghdad, Iraq

## Abstract

The application of phase change materials (PCMs) in green buildings has been increasing rapidly. PCM applications in green buildings include several development models. This paper briefly surveys the recent research and development activities of PCM technology in building applications. Firstly, a basic description of phase change and their principles is provided; the classification and applications of PCMs are also included. Secondly, PCM models in buildings are reviewed and discussed according to the wall, roof, floor, and cooling systems. Finally, conclusions are presented based on the collected data.

## 1. Introduction

Thermal energy storage (TES) is classified as one of the main key technologies for energy storage in the future. Various types of TES technologies exist, such as sensible heat TES, which stores heat in fluid or solid form, and latent heat TES, which uses latent heat during the phase change process and in thermoelectric devices, chemical energy, photochemical reactions, and different concentrations. TES can rapidly release or store large amounts of heats because solar energy and heat are intermittent heat sources [1]. TES is an attractive technology because it is the most appropriate method to correct the gap between the demand and supply of energy [2]. In recent years, most research has investigated phase change materials (PCMs) by studying different commercial PCMs with different melting temperatures.

Rezaei et al. [3] investigated the effect of different PCMs with different melting temperatures on energy and exergy efficiencies by considering the price of energy and exergy for each PCM type. Li et al. [4] established an analytical temperature model based on the lumped parameter method to obtain the enthalpy difference function and calculate the enthalpy difference of composite materials made of two types of inorganic salts. Their results showed that the melting point and enthalpy difference of the binary eutectic LiNO_3_–NaNO_3_, LiCL–NaCL, and Li_2_CO_3_–Na_2_CO_3_ that determined by an analytical temperature model based on the lumped parameter method were consistent with the results from standard methods. In the current study, the review of development surveys of PCM technology for building applications is conducted.

## 2. PCMs and Their Classifications

**Figure 1:**
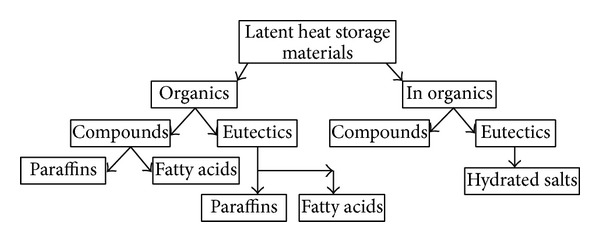
Classifications of phase change materials.

Latent heat storage materials are considered PCMs. Numerous researchers have presented the classifications of PCMs (Figure 1) [5]. The PCMs used in the design of a thermal storage system should have desirable chemical, kinetic, and thermophysical properties [6]. The main characteristics required for good PCMs are presented in Table 1.

## 3. Applications of PCMs

The following are the different PCM applications:

- domestic hot water tanks [7, 8]: according to the literatures, for domestic hot water applications the phase change melting temperature should be around 60°C;
- heat transfer [9, 10]: different PCMs used in different heat exchanger configurations to enhance the heat transfer in system;
- space cooling and heating in buildings and building energy conversation [11–13]. The PCM will break up the rising of ambient temperature where by the material will change from solid to liquid. The applications for examples: PCM wall, ceiling and gypsum boards, trombe wall, and floor heating;
- solar energy utilization [12–14]. Cascaded latent heat storages for stem generation and concentric solar power plant;
- peak load shifting [15]. There are three types of cold storage systems (chilled water storage system, ice storage system and eutectic salt storage system) that used as an effective mean of shifting peak electrical load;
- industrial applications [9, 12, 13, 16]. Thermal protection of food: transport, hotel trade and ice cream;
- ice storage, transport of temperature-sensitive materials, and air-conditioning systems [2, 12, 13];
- thermal insulation for functional fibers [9, 10, 12, 13];
- TES at high temperature [17–19]. PCMs with melting temperature above 300°C (pure salts, metal eutectics, and salt eutectics) have the potential for thermal energy storage in concentrated solar power plant.

## 4. Modeling of PCM Integrated Buildings

TES systems with PCMs can reduce two present problems, namely, the use of fossil fuels and the environmental impacts of global warming. TES plays a crucial role in a wide variety of industrial, commercial, and residential applications. The use of TES with PCMs in buildings can enhance human comfort by decreasing the frequency of the internal air temperature fluctuation. Thus, the indoor air temperature is close to the desired temperature for an extended period. According to available literature, several promising developments are handled by using TES with PCMs in buildings. The survey of these developments is briefly explained below.

### 4.1. PCMs with Wall System

Phase change materials technology is playing an increasing rule in building applications. As reported in literatures, the use of PCMs in buildings is classified as follows.

#### 4.1.1. PCM as Middle Layer in Wall

**Figure 2:**
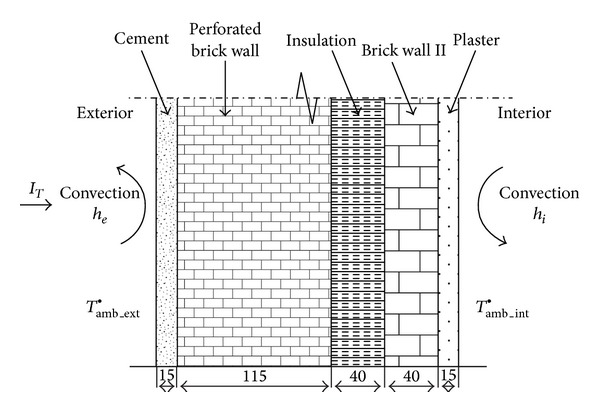
A schematic diagram showing the wall layers of the typical external wall (base composite wall) used in the simulations.

Models for various designs have been developed to study the performance of PCM integration in building walls. Romero-Sánchez et al. [20] evaluated the incorporation of PCMs in natural stone. Experimental and computational studies were conducted to improve the thermal properties of natural stone by exploiting the associated latent heat storage phenomenon. Experimental techniques were then used to construct concrete pilot houses. The pilot houses were covered with transventilated facade designs by using the “Spanish Bateigazul” natural stone. The results showed that a smooth indoor temperature profile is obtained when PCMs are implemented. An improvement in human comfort and a reduction in energy consumption can be anticipated. Izquierdo-Barrientos et al. [21] studied the influence of PCMs in external building walls. Different external building wall configurations were analyzed for a typical building wall by varying the PCM layer location, ambient conditions, wall orientation, and PCM phase transition temperature.  Figure 2 shows the schematic of the wall. This wall represents standard Spanish construction and consists of a first cement layer (15 mm thick) followed by two layers of brick wall (115 and 40 mm thick) with a 40 mm layer of insulation between the brick layers. Finally, a 15 mm plaster layer is placed on the interior of the building. A 1D transient heat transfer model was numerically developed and solved by using a finite difference technique. No significant reduction was observed in the total heat lost during winter regardless of the wall orientation or PCM transition temperature. Significant differences were observed in the heat gained during the summer period because of the elevated solar radiation fluxes.

**Figure 3:**
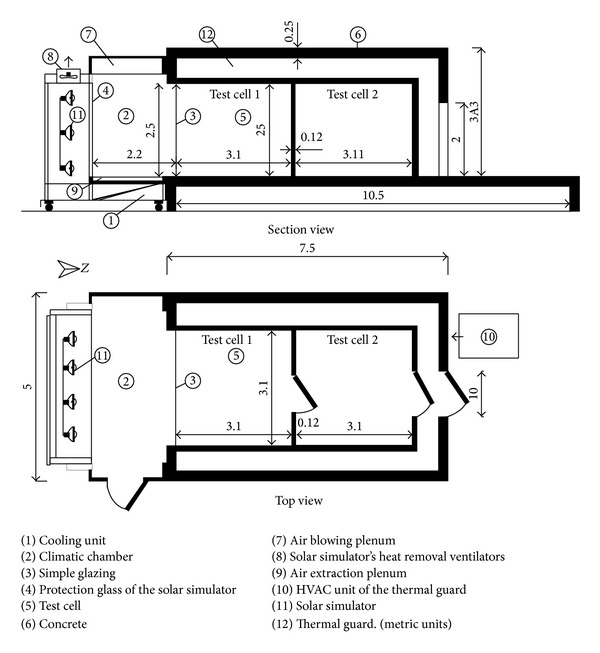
Scheme of the experimental setup.

Kuznik and Virgone [22] experimentally analyzed the comparative thermal performances of a PCM copolymer composite wallboard. The test room was composed of two identical enclosures called Test Cells 1 and 2 (Figure 3). The test cell had a volume of 3.10 m × 3.10 m × 2.50 m and was bounded on five sides by air volumes regulated at a constant temperature. The sixth face was a glazed facade that isolated the test cell from a climatic chamber. The results showed that the air temperature in the room with PCM decreases up to 4.2°C. Comfort enhancement is important if surface temperatures are considered, and the PCM wallboards enhance the natural convection in the room. Furthermore, no thermal stratification exists in the room with the PCM copolymer composite wallboard compared with the room without the composite. Chan [23] assessed the thermal and energy performance of a residential building with PCM integrated external walls in the living room and bedroom. A typical residential flat without a PCM wallboard was used as the base case for comparison. The computer simulation results showed that the living room of a residential flat with a west-facing integrated external wall provides a comparatively significant decrease in interior surface temperature up to a maximum of 4.14%. An annual energy saving of 2.9% was achieved for an air-conditioning system, and the energy payback period was estimated to be 23.4 years.

**Figure 4:**
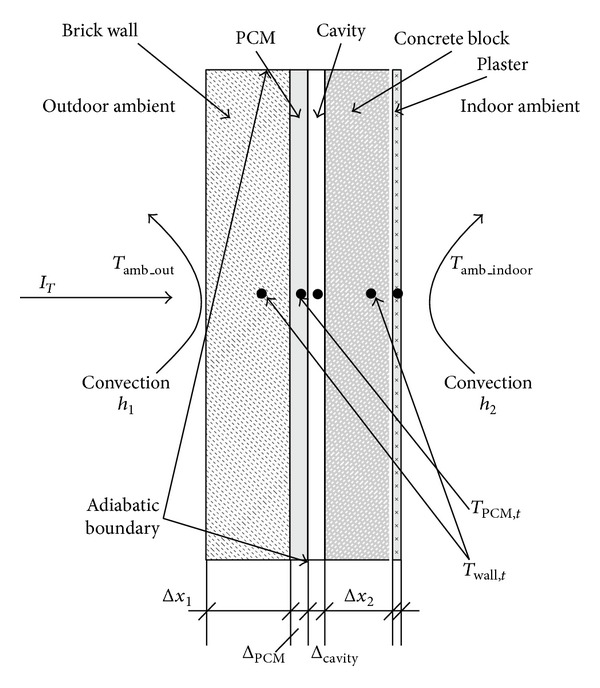
A schematic diagram illustrating the heat transfer in a PCM augmented wall.

**Figure 5:**
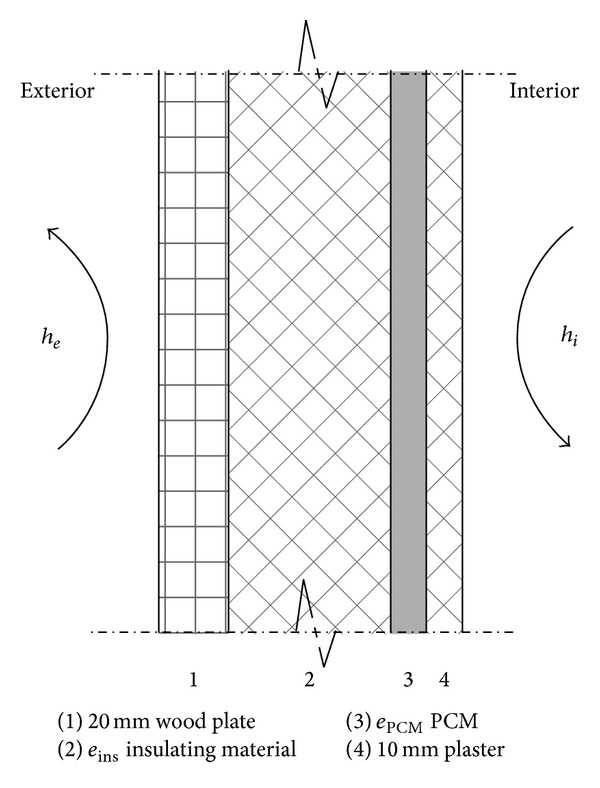
Test wall composition.

Zwanzig et al. [24] investigated the 1D transient heat equation via the multilayered building envelope to study the energy saving potential of PCM for residential homes. In this study, a PCM composite wallboard incorporated into the walls and roof of a typical residential building across various climate zones was examined. The simulation results showed that the optimal location for PCM placement within the building envelope depends on the resistance values between the PCM layer and the exterior boundary conditions. By contrast, the PCM composite wallboard can reduce the energy consumption in summer and winter and can shift the peak electricity load in the summer. Mirzaei and Haghighat [25] proposed a new and fast 1D analytical model for PCM-TES applications in building simulation programs. The accuracy of a resistor-capacitor (RC) circuit model concept that contains variable capacities for the resistor and capacitor significantly depends on the number of synchronized RC circuits. Huang et al. [26] summarized the results of the theoretical investigation and analysis of the temperature regulation effects resulting from the incorporation of PCMs in a building cavity wall. Various quantities of different PCM materials with phase change temperatures of 28 and 43°C were incorporated into a selection of wall constructions. The PCMs were assumed to be directly attached to the surfaces of the masonry wall (Figure 4).

de Gracia et al. [27] evaluated the environmental impact of using PCMs in a typical Mediterranean building. Three hypothetical scenarios were proposed and studied by using the life cycle assessment (LCA) process to highlight the critical issues: different temperature control systems, different PCM types, or different weather conditions. The results showed the following. (1) The addition of PCM in the building envelope decreases energy consumption during building operations but does not significantly reduce the global impact throughout the lifetime of the building. (2) The use of hydrated salts (SP-25 A8 in this study) presents a manufacturing impact that is approximately 75% lower than paraffins (RT-27). (3) The LCA for the real cubicles shows an impact reduction of 37% when disposal polyurethane (PU) is added to the reference cubicle (REF). Kuznik et al. [28] investigated the optimal value of the PCM wallboard thickness. The PCM wallboard was used in lightweight buildings to reduce room air temperature fluctuations.  Figure 5 represents a complete description of the test wall. The wall tested was composed of 2 cm of outside wood, a variable insulating material (glass wool) with a thickness of 10 cm and variable PCM thickness, and 1 cm of plaster. The in-house numerical code CODYMUR was developed to calculate the optimal thickness value. The results showed that an optimal value exists according to daily external and internal temperature fluctuations.

#### 4.1.2. PCM as Internal Layer in Wall

**Figure 6:**
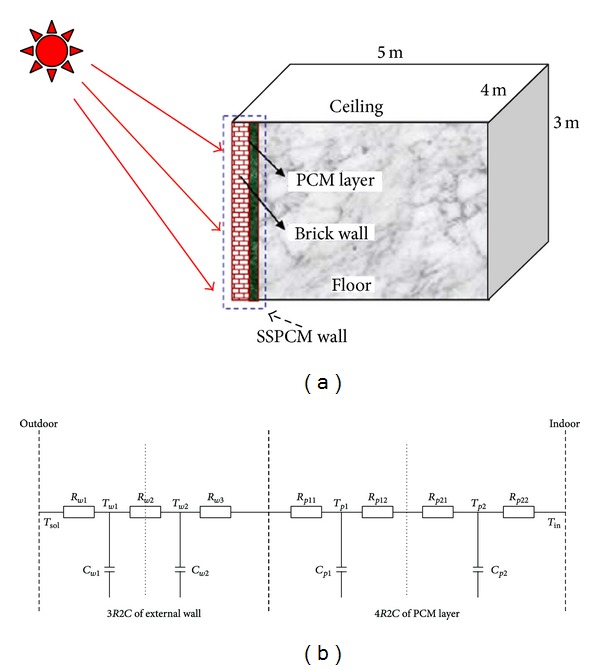
(a) Schematic of SSPCM wall and the ideal model house (b) Schematic of the simplified dynamic building model.

The thermodynamic models of building structures by using PCMs were presented by [29] to analyze their effects on building energy performance at different conditions. A simplified physical dynamic model of building structures integrated with shape-stabilized PCM (SSPCM) was developed and validated. The simplified physical model represents the wall by three resistances and two capacitances and represents the PCM layer by four resistances and two capacitances (Figure 6). The key issue for this model is parameter identification. A few PCM models with detailed physics and good accuracy in simulating the thermodynamic behavior of building structures were integrated with PCM layers. Validation results showed that the simplified model can accurately represent light walls and median walls integrated with SSPCM.

**Figure 7:**
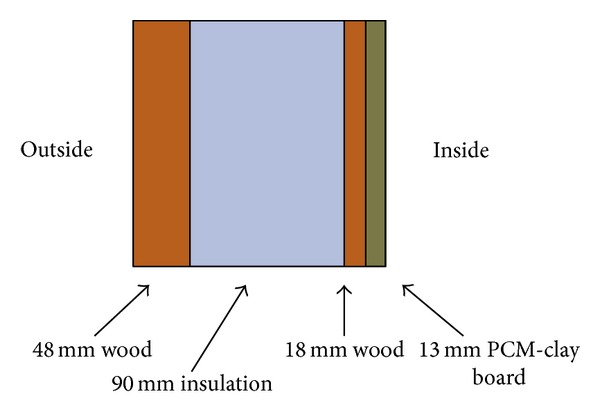
Experimental wall construction.

Computational fluid dynamic simulation was used to evaluate the effectiveness of PCM clay wallboards to reduce peak indoor temperatures of non-air-conditioned spaces in the summer months [30]. The results showed that PCM clay wall boards can reduce the peak temperature of indoor spaces by 3 K compared with conventional traditional plasterboards and can prevent overheating in the summer months. The performance of the clay boards depends on the PCM quantity, building fabric characteristics, and internal and external heat gains. By contrast, a test cell with internal dimensions of 1.3 m × 0.8 m × 1.4 m with a glazed facade dimension of 1.3 m × 0.8 m was constructed to provide a controlled environment where in the transient behavior of air and PCM could be investigated. The wall/ceiling/floor structure was made of 48 mm plywood, 90 mm insulation, and 18 mm plywood with skimmed PCM clay boards placed on the inside surface of the walls only (Figure 7).

**Figure 8:**
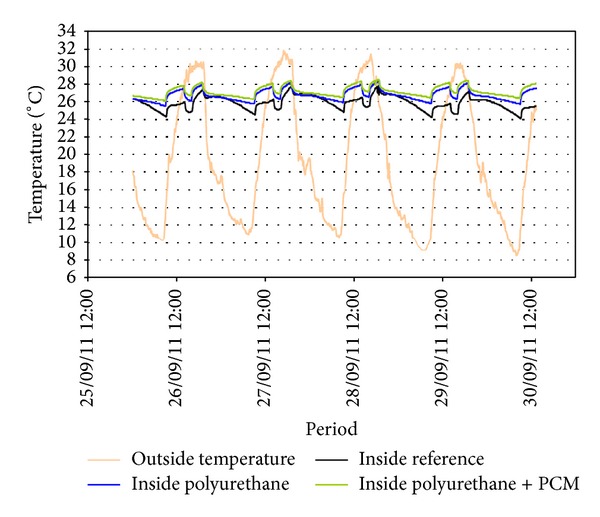
Free floating with internal loads (office profile): inside and outside temperatures.

Lidia et al. [31] analyzed experimentally the PCM performance in a scenario with internal thermal gains. Three different cubicles with the same internal dimensions (2.4 m × 2.4 m × 2.4 m) located in Puigverd de Lleida, Spain, were used to perform the experiment. These cubicle systems were constructed as follows: (1) the REF was built by a traditional brick system that is based on two layers of bricks with an air gap and without insulation; (2) PU cubicle was built by a traditional brick system but with 5 and 3 cm of spray foam PU in the walls and in the roof, respectively; (3) the PCM cubicle was built in the same manner as the previous cubicle but with a PCM layer in the southern and western walls and on the roof. CSM panels that contain RT-27 paraffin (Rubitherm) were located on the internal side of the PU. The results of the summer period showed that the PCM cubicle stores the heat produced by the internal loads, thus limiting the heat dissipation to the outer environment. The REF had higher temperature fluctuations in its envelope (27.5°C to 24°C) than other cubicles with insulation (28°C to 26°C) (Figure 8).

**Figure 9:**
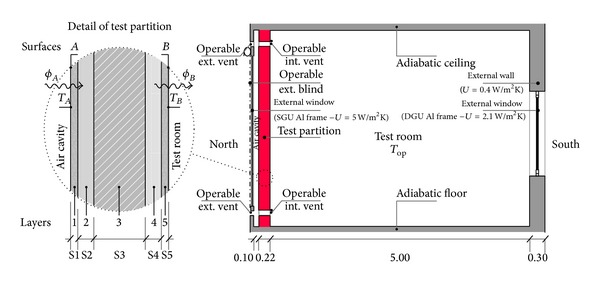
Test room section and details of test partition: (1–5) Temperature test partitions (S1 and S5) Plasterboard panel (S2 and S4) PCMs and (S3) Mineralized wood panel.

Francesco [32] summarized his research to provide a comparative assessment of thermal comfort variability derived from several PCMs integrated into the internal partition of a Trombe wall and exposed to cold, mild, and hot climates. A simple test room (square plant of 5 m × 5 m) with two walls exposed to the north and south was modeled and simulated by using Energy-Plus software. The south wall was modeled as an insulated lightweight wall (own weight less than 100 kg/m^2^) with a double-glazed window.  Figure 9 shows the schematic section of the modeled test room. The results showed that in mild cold and temperate climates, the integration of PCMs on the outside surface of the intermediate partition of a Trombe wall produced an optimal reduction in the fluctuations of inside temperatures, which remain stable at comfortable values.

**Figure 10:**
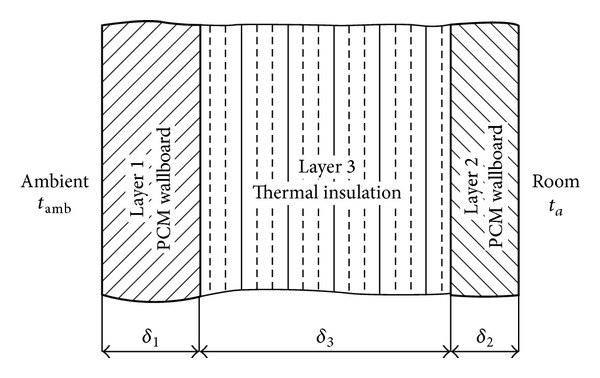
The structure of the composite PCM wallboard wall system.

Two identical rooms were tested by Kuznik et al. [33] to assist PCM wallboard use for the renovation of a tertiary building. One room was equipped with PCM wallboards in the lateral walls and in the ceiling and another room was not equipped. They showed that the PCM wallboards enhance the thermal comfort of occupants due to air temperature and radiative effects of the walls. Diaconu and Cruceru [34] proposed a new type of composite wall system that incorporates PCMs. This new wall system consists of three functional layers denoted by numbers one to three (Figure 10). The outer layers consist of a building material that is integrated with a PCM, and the middle layer consists of conventional thermal insulation. The enthalpy method was used to account for the variable thermophysical properties of the PCM wallboards. They observed that the outer PCM wallboard layer prevents an excessive increase in the temperature of the insulation interface. This effect occurs for a range of melting point values of the PCM incorporated into Layer 1.

**Figure 11:**
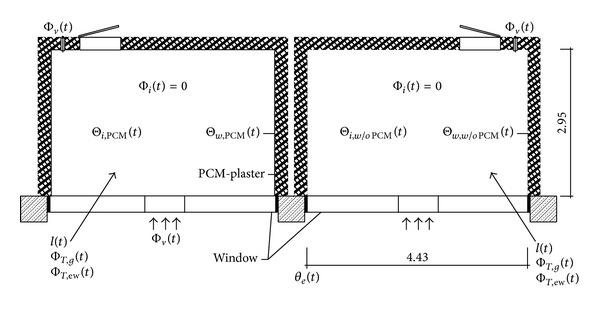
Test rooms (on the left with PCM plaster).

The effect of plaster and salt mixture on room temperature reduction was studied [35]. An experimental setup that consisted of two identical test rooms was built near Weimar, Germany (Figure 11). The initial thickness of the plaster coating was 1 cm and later increased to approximately 3 cm. The plastered surface area per room was 52 m^2^. During the measurements, additional tubes filled with a modified CaCl_2_–6H_2_O were introduced to improve the thermal effect in the PCM-conditioned room. The results showed a 4 K reduction in the peak temperature. Furthermore, the results proved that PCMs lose their heat storage capacity after consecutive hot days.

**Figure 12:**
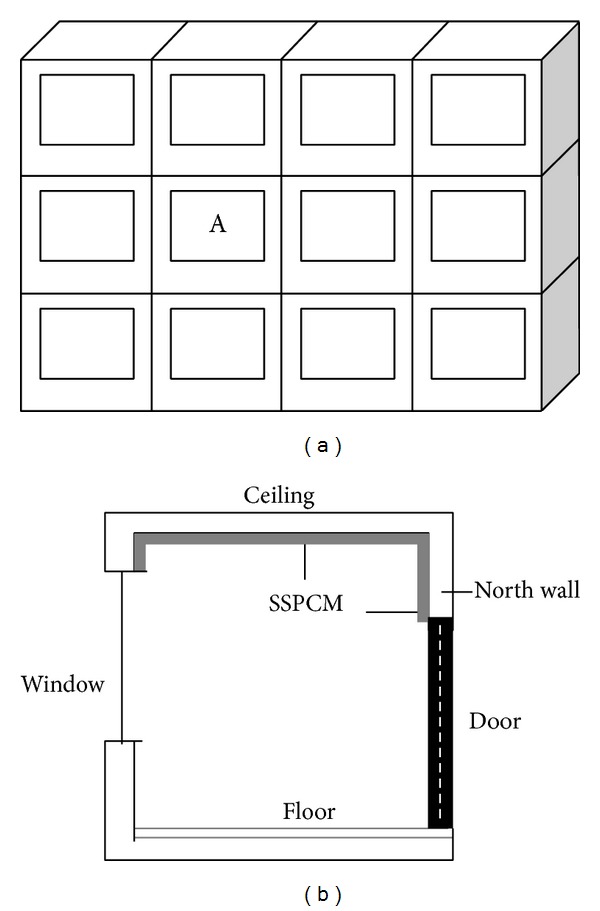
Schematic of the simulated room: (a) location of the simulated room A in the building and (b) profile of the room A with SSPCM.

Zhou et al. [36] performed a numerical analysis on the thermal effect of SSPCM plates as inner linings were subjected to indoor air temperature under night ventilation conditions in summer. A building in Beijing that had SSPCM plates as inner linings of walls and the ceiling was considered for analysis (Figure 12). The results showed that the SSPCM plates decrease the daily maximum temperature by up to 2°C because of the cool storage at night.

#### 4.1.3. PCM with Wall and Air-Conditioning System

Quantitative studies were conducted by [37] to investigate the effects of SSPCM and different control strategies on energy consumption and peak load demand. An office building (i.e., a 46-storey commercial building in Hong Kong) that used a typical variable air volume air-conditioning system was selected and simulated as the reference building. An office floor with approximately 2400 m^2^ of floor area was studied. The offices and associated air-conditioning system in 1/2 of the floor (i.e., 1200 m^2^) were selected. The simulated floor consisted of eight open-plan offices (zones). The external walls were concrete with thicknesses of 115 mm, whereas the internal walls were brick. The envelopes were enhanced by integrating the SSPCM layers into the walls, whereas the air-conditioning system and other building configurations remained unchanged. The results showed that the use of SSPCM in the building significantly reduces building electricity costs (over 11% in electricity cost reduction and over 20% in peak load reduction). Aranda-Usón et al. [38] used Life cycle assessment to determine if energy savings are large enough to balance the environmental impact caused by PCM manufacturing and PCM installation on tiles. This study evaluates the two main aspects of PCM introduction in buildings: the energy savings obtained compared with the energy consumption for air conditioning when PCMs are not applied in the building; the environmental impact associated with PCM inclusion. A total of 15 different case studies are analyzed by combining 3 different PCMs applied to 5 Spanish weather conditions. The main results conclude that the use of PCM reduces the overall energy consumption and environmental impact.

### 4.2. PCMs with Roof System

**Figure 13:**
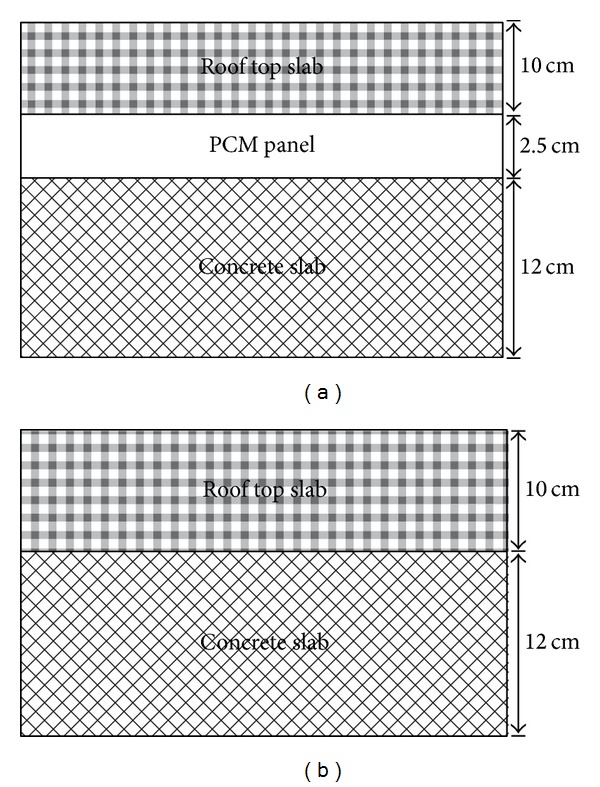
Cross-sectional view of the roof (a) with PCM panel and (b) without PCM panel.

Pasupathy et al. [39] analyzed and discussed theoretical and experimental investigations to study the thermal performance of an inorganic eutectic PCM-based thermal storage system for energy conservation in buildings. An experimental setup that consisted of two identical test rooms (1.22 m × 1.22 m × 2.44 m) was constructed. One room did not have PCM on the roof, whereas the other room had a PCM panel between the bottoms concrete slab and rooftop slab. The inner walls except the ceiling of the rooms were insulated by 6 mm-thick plywood with on all sides to study the sole effect of PCM panel on the roof. The PCM panel was made up of 2 m × 2 m stainless steel with a thickness of 2.54 cm; the stainless steel accommodated inorganic salt hydrate (48% CaCl_2_ + 4.3% NaCl + 0.4% KCl + 47.3% H_2_O) as PCM. During the experiment, the measured room temperatures varied at approximately 27 ± 3°C. The same experiment setup in [39] was used to study the effect of double-layer PCM in a building roof. One room was constructed without PCM on the roof to provide a reference case for comparison with the experimental room that included the PCM [40].  Figure 13 represents the cross-sectional views of the roof with and without PCM. A mathematical model was developed by using the finite volume method to predict the thermal behavior of a roof with PCMs. The results showed that the environment has an insignificant effect on the inner surface of the concrete slab because all the heat energy is absorbed by the PCM in the roof. By contrast, a significant fluctuation was observed in the ceiling of the non-PCM room because of the immediate influence of the external environment.

### 4.3. PCMs with Floor Heating System

Some researchers have been focusing on PCMs with floor heating system. Mazo et al. [41] described a one-dimensional finite difference scheme to simulate a radiant floor system with PCM in simple building types. The effective capacity method was used for PCM simulation. They investigated that the radiant floor with PCM was able to meet the heating demand requirements with a practically total shift of the electric energy consumption from the peak period to the off-peak electricity demand time. A numerical model was created by Arnault et al. [42] to determine the thermal performance of internal surfaces, including PCM, and to compare a typical concrete floor with a PCM floor. The PCM floor was composed of different materials. Three different objective functions were used to define the thermal performance, such as parametric studies, to understand the influence of the thickness of a typical concrete floor, the optimization of the melting temperature, and the thickness and position of a PCM. The results showed that the thickness of the concrete floor can be optimized based on the three criteria. Furthermore, floor performance is enhanced by the inclusion of a PCM layer.

### 4.4. PCMs with a Building Cooling System

**Figure 14:**
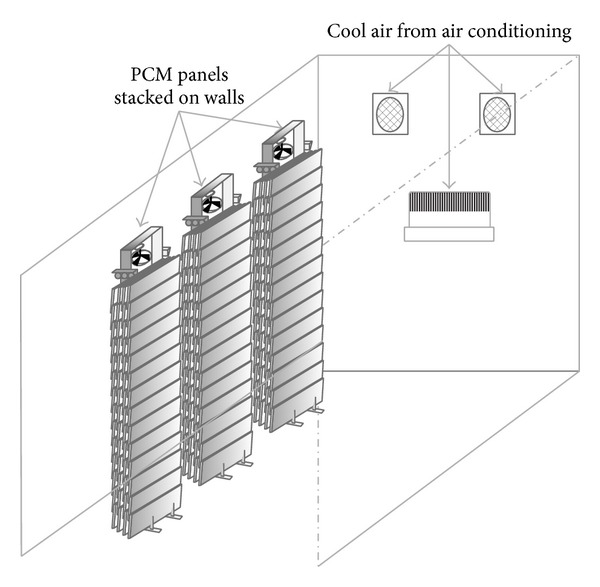
Schematic view of high-density polyethylene panel based TMS in the test room.

Tyagi et al. [43] experimentally designed and studied the thermal management system (TMS) to reduce the use of air-conditioning systems in buildings. A prototype test room and a TMS for cooling applications in the building were designed to investigate the performance of TMS with different operation conditions. Furthermore, the TMS consisted of CaCl_2_–6H_2_O as PCM, 60 rectangular-shaped high-density polyethylene panels as a heat exchanger/container, 3 stacks for panels, 3 fans for air circulation, 1 air-conditioning system of 1.5 TR (Figure 14), and 3 different heat sources (1000, 2000, and 3000 W). The results of the experimental study showed that the temperature profiles of the test rooms with room heaters were shortest (3 kW room heater) and longest (1 kW room heater) with and without TMS, respectively. Over a 24 h period, the variation of the room and ambient air temperatures vary from 19°C to 32°C and 18°C to 34°C, respectively.

Experiments with a DOE strategy were conducted by [44] to study the application of PCMs in free-cooling systems to store cool outdoor air during the night and supply indoor cooling during the day. An experimental setup was designed and constructed to allow the performance testing of PCM under the main influence parameters: energy/volume ratio in the encapsulation, load/unload rate of the storage, and installation cost. The system consisted of a closed-air circuit with fans, heating and cooling devices, and thermal energy. The statistical analysis showed that the thickness of the encapsulation, airflow, inlet temperature of the air, and interaction thickness/temperature have significant influences on the solidification process. The inlet air temperature has a higher influence than the thickness of the encapsulation.

**Figure 15:**
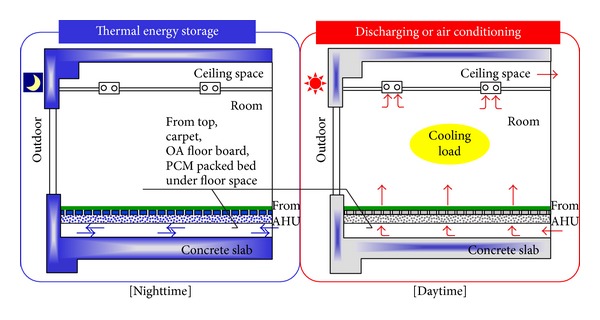
Concept of the system.

A new floor-supply air-conditioning system was proposed in [45] by using PCM to augment the mass thermal storage of buildings. A small experimental system was constructed (Figure 15), and the body of the apparatus was constructed from 100 mm-thick heat-insulating material. The interior was 1000 mm long and 500 mm deep, thus providing a floor area of 0.5 m^2^. The cold storage bodies were concrete plates as concrete slabs, OA floorboards with numerous holes, and a PCM-packed bed. The measurement results indicated that 89% of the daily cooling load can be stored each night in a system that uses a 30 mm-thick packed bed of granular PCM.

Persson and Westermark [46] evaluated a comfort cooling strategy to attain good indoor climate during summer in a Swedish Passive House while maintaining good energy efficiency by using a PCM air-heat exchanger. A Matlab code was used to analyze the climate files and the thermodynamic properties of PCM storage. Thereafter, Energy-Plus software was used to build the simulations. The results showed that the PCM can remove a substantial amount of degree hours with excessive temperatures.

## 5. Conclusions 

A review of the development of PCM models in buildings is prepared to survey literature that have reported various models of PCMs in building components (e.g., walls, roof, and walls and roof). The following conclusions are drawn based on the analysis of literature on PCM technology in building applications:

- the thermal improvements in buildings caused by the integration of PCMs depend on the type and melting temperature of PCMs, the components of the building (wall or roof), the orientation and design of the building, and the climate;
- all of the PCM models reviewed have good potentials to reduce heating and cooling loads by enhancing the storage capacity of the building envelope;
- on the basis of the review of previous research, several models are used to study the effects of PCM technology in building walls and building cooling.

## Figures and Tables

**Table 1:** Properties of phase change materials.

Properties			
Thermophysical	Nucleation and crystal growth	Chemical	Economics
(i) Melting temperature (ii) High latent heat of fusion (iii) High specific heat (iv) High thermal conductivity (v) Small volume change on phase transformation and small vapor pressure (vi) Congruent melting of the PCM for a constant storage capacity (vii) Reproducible phase change	(i) High nucleation rate to avoid sub cooling of liquid phase (ii) High rate of crystal growth	(i) Complete reversible freeze/melt cycle (ii) No degradation after a large number of freeze/melt cycle (iii) No corrosiveness (iv) Nontoxic, (v) Non flammable (vi) Nonexplosive	(i) Abundant (ii) Available (iii) Cost effective (iv) Easy recycling and treatment (v) Good environmental performance
